# Acceptability of Condoms, Circumcision and PrEP among Young Black Men Who Have Sex with Men: A Descriptive Study Based on Effectiveness and Cost

**DOI:** 10.3390/vaccines2010129

**Published:** 2014-02-18

**Authors:** Richard A. Crosby, Angelica Geter, Ralph J. DiClemente, Laura F. Salazar

**Affiliations:** 1 Department of Health Behavior, College of Public Health, University of Kentucky, 121 Washington Ave, Lexington, KY 40506, USA; E-Mail: crosby@uky.edu; 2 Department of Behavioral Sciences and Health Education, Rollins School of Public Health, Emory University, Atlanta, GA 30322, USA; E-Mail: rdiclem@emory.edu; 3 School of Public Health, Georgia State University, One Park Place, Suite 714, Atlanta, GA 30303, USA; E-Mail: lsalazar1@gsu.edu

**Keywords:** African American men who have sex with men, HIV/AIDS, condoms, PrEP, circumcision

## Abstract

The current study examined and compared the willingness of young Black men who have sex with men (YBMSM) to accept pre-exposure prophylaxis (PrEP), adult male circumcision, and condoms for reducing their risk of HIV acquisition. The majority (67%) reported unprotected receptive anal sex in the last six months. About three-quarters (71%) would accept using PrEP if it was 100% effective. Cost influenced PrEP acceptance with 19% indicating acceptance at $100 per month co-pay. Of those not circumcised, 50% indicated willingness if circumcision was 100% effective. Acceptance of circumcision decreased markedly to 17% with co-pays of $100. About 73% of men were willing to use condoms if they were 100% effective and 50% indicated a willingness at the cost of $10 per month. The findings suggest that condom use promotion strategies should remain at the forefront of public health efforts to control HIV incidence among YBMSM.

## 1. Introduction

The southern region of the United States comprises the largest percentage (18%) of Black Americans and accounts for 46% of all new HIV diagnoses and more than 55% of HIV prevalence [1]. Young Black men who have sex with men (YBMSM) represent 73% of HIV incidence among all Black men and 37% of all MSM [2,3]. YBMSM aged 13–29 years are the only subgroup to have experienced a continuous increase in HIV incidence rates during the last three years [4]. Based on these marked racial/ethnic disparities, many questions remain about the acceptance of newly developed as well as established HIV prevention strategies for YBMSM.

The use and effectiveness of condoms against HIV and other sexually transmitted infections (STI) are empirically supported, showing a 59% decrease in STI acquisition with accurate and consistent condom utilization [5,6,7,8,9]. Despite this effectiveness, continued disparities have increased the need for additional approaches to HIV prevention [10]. In recent years, biomedical strategies have reemerged as promising efforts in this regard.

Pre-exposure prophylaxis (PrEP) is an empirically supported antiretroviral medication consumed prior to HIV exposure to prevent potential acquisition [11]. Results from the PrEP Initiative study showed a 44% reduction in HIV risk transmission among MSM and eventually led to the release of federal guidelines and FDA approval for PrEP as an HIV prevention strategy [12,13,14]. PrEP acceptability has varied between 30% and 80% [15,16,17] and its uptake has been influenced by demographic characteristics (e.g., age and education), sexual risk behavior and perception of risk [18,19].

Another biomedical strategy is adult male circumcision [20,21], which has been recommended as part of a comprehensive approach to HIV prevention [22]. However, effectiveness data have varied among MSM [23,24,25] with a paucity of data specific to Black MSM [26,27].

The continued increase of HIV in YBMSM has created a need to understand what prevention strategies are most acceptable to this population. Therefore, the purpose of this study was to describe the willingness of YBMSM to accept the use of condoms, PrEP, and circumcision for reducing their risk of HIV acquisition.

## 2. Methods

Participants were recruited between 15 January 2013 and 14 February 2013, through banner advertisements on the Black Gay Chat website. These advertisements were restricted to residents of Mississippi, Louisiana, Alabama and Georgia. Website visitors who clicked on a banner ad were redirected to the internet-survey for completion. The survey was developed through the Qualtrics online system and included no accessibility limitations (*i.e.*, desktop or mobile preferences). Young men were eligible if they had sex with a man in the past six months, were 18–39 years of age, and identified as being African American or Black. Incentives were not provided. The survey was anonymous and assessed questions assessing demographics, sexual risk behavior and determinants in the utilization of HIV prevention methods. The Institutional Review Board at the University of Kentucky approved all study protocols. Data were analyzed using frequency distributions.

## 3. Results

The sample consisted of young Black men who have sex with men (YBMSM) (*N* = 95), ages 18–39 years (mean = 26.8, SD = 5.66). In the last six months, 72% reported insertive anal sex and 74% reported receptive anal sex. The majority (71%) reported engaging in at least one act of anal sex that was not condom-protected. During the last six months, 67% reported at least one instance of engaging in unprotected receptive anal sex and 56% reported engaging in one instance of unprotected insertive anal sex (See Table 1).

**Table 1:** Characteristics of the Study Sample, African American Men, Aged 18–39 (*N* = 95).

Variable	Mean (SD)	n (%)
Age	26.8 (5.66)	
Anal insertive (top) sex		
Yes		68 (71.6)
No		27 (28.4)
Anal insertive (top) sex with a condom		
Yes		50 (86.2)
No		8 (13.8)
Anal insertive (top) sex without a condom		
Yes		33 (55.9)
No		26 (44.1)
Anal receptive (bottom) sex		
Yes		70 (73.7)
No		25 (26.3)
Anal receptive (bottom) sex with a condom		
Yes		49 (79.0)
No		13 (21.0)
Anal receptive (bottom) sex without a condom		
Yes		42 (66.7)
No		21 (33.3)

Nearly three-quarters (71%) of the men were willing to accept PrEP if it was 100% effective. Willingness to accept this method decreased with a lower level of effectiveness: 75% effectiveness (43%) and 50% effectiveness (21%). Cost had an influence on men’s willingness to accept PrEP: 19% were willing to accept the medication with a personal cost of $100. Table 2 provides greater details.

The majority (75%) of the participants were circumcised. Of those young men who were not circumcised (*n* = 24), 50% indicated a willingness to be circumcised if this procedure was 100% effective in avoiding HIV infection. Acceptance of circumcision as an HIV prevention strategy decreased markedly to 17% with a personal cost of $100. Table 3 provides more information regarding the decline in acceptance based on cost and effectiveness.

**Table 2:** Acceptance of pre-exposure prophylaxis (PrEP) as a Safe Sex Measure (*N* = 95).

Variable	n (%)
PrEP acceptance based on 100% effectiveness against HIV	
Yes	67 (70.5)
No	28 (29.5)
PrEP acceptance based on 75% effectiveness against HIV	
Yes	41 (43.2)
No	54 (56.8)
PrEP acceptance based on 50% effectiveness against HIV	
Yes	20 (21.1)
No	74 (77.9)
PrEP acceptance based on cost-Free	
Yes	58 (61.1)
No	36 (37.9)
PrEP acceptance based on cost-$100 or less per month	
Yes	18 (18.9)
No	77 (81.1)
PrEP acceptance based on cost-$500 per month	
Yes	16 (16.8)
No	77 (81.1)
PrEP acceptance based on cost-$1000 per month	
Yes	13 (13.7)
No	82 (86.3)

**Table 3:** Acceptance of Circumcision as a Safe Sex Measure (*N* = 24).

Variable	n (%)
Circumcised	
Yes	71 (74.7)
No	24 (25.3)
Circumcision acceptance based on 100% effectiveness against HIV	
Yes	12 (50.0)
No	12 (50.0)
Circumcision acceptance based on 75% effectiveness against HIV	
Yes	9 (37.5)
No	15 (62.5)
Circumcision acceptance based on 50% or less effectiveness against HIV	
Yes	8 (33.3)
No	16 (66.7)
Circumcision acceptance based on cost-Free	
Yes	13 (54.2)
No	11 (45.8)
Circumcision acceptance based on cost-$100 or less	
Yes	4 (16.7)
No	20 (83.3)
Circumcision acceptance based on cost-$500 or more	
Yes	1 (4.2)
No	23 (95.8)

The majority of the men (73%) were willing to use condoms if they were 100% effective, with 50% indicating this willingness to accept this prevention strategy at a cost of $10 per month. Table 4 provides greater detail about these findings. 

**Table 4:** Acceptance of condoms as a safe sex measure (*N* = 95).

Variable	n (%)
Condom acceptance based on 100% effectiveness against HIV	
Yes	69 (72.6)
No	26 (27.4)
Condom acceptance based on 75% effectiveness against HIV	
Yes	57 (60.0)
No	38 (40.0)
Condom acceptance based on 50% or less effectiveness against HIV	
Yes	40 (42.1)
No	55 (57.9)
Condom/Lubricant acceptance based on cost-Free	
Yes	64 (67.4)
No	31 (32.6)
Condom/Lubricant acceptance based on cost-$10 per month	
Yes	47 (49.5)
No	48 (50.5)
Condom/Lubricant acceptance based on cost-$100 per month	
Yes	18 (18.9)
No	77 (81.1)

## 4. Discussion

Regardless of the HIV prevention method being offered, small personal costs have a substantial adverse influence on acceptance of PrEP, circumcision or condom use. Generally, the level of acceptance for all three methods was low, unless the method was rated at 100% efficacy and provided at minimal cost to the participant. The findings suggest that even under ideal circumstances (100% effective and free) a large proportion of men may not be willing to use any of these methods. This observation led to a post-hoc analysis that calculated the percent of men who would not accept the method even under both ideal circumstances (100% efficacy and free). This analysis was achieved through the use of a contingency table. These findings showed that 27% would not accept PrEP, 42% of those not circumcised would refuse do so, and 21% would not use condoms. These values are high given that the ideal circumstances are unlikely to exist, with the possible exception of condom use.

Findings regarding PrEP are particular intriguing. The current findings are similar to those from other studies that examined barriers to PrEP acceptance [18]. Previous studies have shown that government funding to assist in the accessibility of PrEP could be a facilitator to the acceptance of this HIV prevention method [18]. Cost-effectiveness has been one of the primary considerations in the use of public funds for these prevention strategies. Delivery of PrEP was found to be a cost-effective strategy for high-risk populations [28,29], but acceptance among YBMSM may alter this equation. Resources to assist in subsidizing personal costs to YBMSM may be needed to enhance uptake of these prevention strategies [30]. PrEP effectiveness has been established from clinical trials when combined with condom use, HIV testing and other established prevention methods [15,30,31]. Although the current evidence supports this strategy, further research is needed regarding whether YBMSM most at-risk of HIV will indeed seek out a provider to give them PrEP at a price they can afford.

## 5. Limitations

These findings are limited based on the validity of self-reported data. The participants were a sample of men who opted into an online banner-ad survey and therefore the findings are subject to selection bias. Convenience sampling and restrictions to the southern region of the U.S. limits the generalizability of the findings to other populations of MSM. The results are based on a small sample size and therefore further research is warranted. Additionally, the findings provide limited insight to the participants’ knowledge of HIV prevention methods. This information could be a facilitator or barrier to their decision to prefer certain safe sex methods and should be further examined in future research.

## 6. Conclusions

Biomedical approaches to HIV prevention, such as the use of PrEP and circumcision, will ultimately require patient acceptance. Availability alone may not be an adequate response. Given optimal circumstances (*i.e.*, 100% effective and no personal costs) PrEP and circumcision are less acceptable to YBMSM than condom use. Because these optimal circumstances may never exist, findings suggest that condom use promotion strategies should remain at the forefront of public health efforts to control HIV incidence among YBMSM. Further, the study findings suggest that HIV preventive measures offered to YBMSM may not be widely embraced, including condom use. Apathy about preventing HIV infection may be a barrier working against efforts to innovatively protect this population. Thus, the role of behavioral science in HIV prevention is one that can complement and enhance emergent biomedical strategies.

## References

[1] Prejean J., Tang T., Hall H.I. (2013). HIV diagnoses and prevalence in the southern region of the united states, 2007–2010. J. Community Health.

[2] Balaji A.B., Oster A.M., Viall A.H., Heffelfinger J.D., Mena L.A., Toledo C.A. (2012). Role flexing: How community, religion, and family shape the experiences of young black men who have sex with men. AIDS Patient Care STDs.

[3] Centers for Disease Control and Prevention (2013). Diagnosed HIV Infection among Adults and Adolescents in Metropolitan Statistical Areas—United States and Puerto Rico, 2010. HIV Surveillance Supplemental Report 2013.

[4] Prejean J., Ruiguang S., Hernandez A., Ziebell R., Green T., Walker F., Hall H. (2011). Estimated HIV incidence in the united states, 2006–2009. PloS One.

[5] Centers for Disease Control and Prevention (2011). Condoms and Stds: Fact Sheet for Public Health Personnel.

[6] Crosby R.A., Bounse S. (2012). Condom effectiveness: Where are we now?. Sex Health.

[7] Warner L., Macaluso M., Austin H.D., Kleinbaum D.K., Artz L., Fleenor M.E., Brill I., Newman D.R., Hook E.W. (2005). Application of the case-crossover design to reduce unmeasured confounding in studies of condom effectiveness. Am. J. Epidemiol..

[8] Warner L., Newman D.R., Austin H.D., Kamb M.L., Douglas J.M., Malotte C.K., Zenilman J.M., Rogers J., Bolan G., Fishbein M. (2004). Condom effectiveness for reducing transmission of gonorrhea and chlamydia: The importance of assessing partner infection status. Am. J. Epidemiol..

[9] Crosby R.A., Charnigo R.A., Weathers C., Caliendo A.M., Shrier L.A. (2012). Condom effectiveness against non-viral sexually transmitted infections: A prospective study using electronic daily diaries. Sex. Transm. Infect..

[10] Nodin N., Carballo-Dieguez A., Ventuneac A.M., Balan I.C., Remien R. (2008). Knowledge and acceptability of alternative HIV prevention bio-medical products among msm who bareback. AIDS Care.

[11] Baeten J.M., Donnell D., Ndase P., Mugo N.R., Campbell J.D., Wangisi J., Tappero J.W., Bukusi E.A., Cohen C.R., Katabira E. (2012). Antiretroviral prophylaxis for HIV prevention in heterosexual men and women. N. Engl. J. Med..

[12] Thigpen M.C., Kebaabetswe P.M., Paxton L.A., Smith D.K., Rose C.E., Segolodi T.M., Henderson F.L., Pathak S.R., Soud F.A., Chillag K.L. (2012). Antiretroviral preexposure prophylaxis for heterosexual HIV transmission in botswana. N. Engl. J. Med..

[13] Saberi P., Gamarel K.E., Neilands T.B., Comfort M., Sheon N., Darbes L.A., Johnson M.O. (2012). Ambiguity, ambivalence, and apprehensions of taking HIV-1 pre-exposure prophylaxis among male couples in san francisco: A mixed methods study. PloS One.

[14] The U.S. Food and Drug Administration (2012). Fda Approves First Drug for Reducing the Risk of Sexually Acquired HIV Infection.

[15] Grant R.M., Lama J.R., Anderson P.L., McMahan V., Liu A.Y., Vargas L., Goicochea P., Casapía M., Guanira-Carranza J.V., Ramirez-Cardich M.E. (2010). Preexposure chemoprophylaxis for HIV prevention in men who have sex with men. N. Engl. J. Med..

[16] Calderon Y., Jason L., Cowan E., Brusalis C., Mantell J., Sandfort T. (2012). HIV pre-exposure prophylaxis (prep)-knowledge and attitudes among a new york city emergency department patient population. Retrovirology.

[17] Golub S.A., Kowalczyk W., Weinberger C.L., Parsons J.T. (2010). Preexposure prophylaxis and predicted condom use among high-risk men who have sex with men. J. Acquir. Immune Def. Syndr..

[18] Brooks R.A., Kaplan R.L., Lieber E., Landovitz R.J., Lee S.J., Leibowitz A.A. (2011). Motivators, concerns, and barriers to adoption of preexposure prophylaxis for HIV prevention among gay and bisexual men in HIV-serodiscordant male relationships. AIDS Care.

[19] Khawcharoenporn T., Kendrick S., Smith K. (2012). HIV risk perception and preexposure prophylaxis interest among a heterosexual population visiting a sexually transmitted infection clinic. AIDS Patient Care STDs.

[20] Grosskurth H., Mosha F., Todd J., Mwijarubi E., Klokke A., Senkoro K., Mayaud P., Changalucha J., Nicoll A., ka-Gina G. (1995). Impact of improved treatment of sexually transmitted diseases on HIV infection in rural tanzania: randomized controlled trial. Lancet.

[21] Doyle S.R., Calsyn D.A., Ball S.A. (2009). Factor structure of the condom barriers scale with a sample of men at high risk for HIV. Assessment.

[22] World Health Organization (2009). Traditional Male Circumcision Among Young People: A Public Health Perspective in the Context of HIV Prevention.

[23] Wiysonge C.S., Kongnyuy E.J., Shey M., Muula A.S., Navti O.B., Akl E.A., Lo Y.R. (2011). Male circumcision for prevention of homosexual acquisition of HIV in men. Cochrane Database Syst. Rev..

[24] Templeton D. (2010). Male circumcision to reduce sexual transmission of HIV. Curr. Opin. HIV AIDS.

[25] Millett G.A., Flores S.A., Marks G., Reed J.B., Herbst J.H. (2008). Cir cumcision status and risk of HIV and sexually transmitted infections among men who have sex with men: A meta analysis. J. Am. Med. Assoc..

[26] Millett G.A., Ding H., Lauby J., Flores S., Stueve A., Bingham T., Carballo-Dieguez A., Murrill C., Liu K.L., Wheeler D. (2007). Circumcision status and HIV infection among black and latino men who have sex with men in 3 us cities. J. Acquir. Immune Defic. Syndr..

[27] Maulsby C., Millett G., Lindsey K., Kelley R., Johnson K., Montoya D., Holtgrave D. (2013). HIV among black men who have sex with men (msm) in the united states: A review of the literature. AIDS Behav..

[28] Gomez G.B., Borquez A., Case K.K., Wheelock A., Vassall A., Hankins C. (2013). The cost and impact of scaling up pre-exposure prophylaxis for HIV prevention: A systematic review of cost-effectiveness modelling studies. PLoS Med..

[29] Eisingerich A.B., Wheelock A., Gomez G.B., Garnett G.P., Dybul M.R., Piot P.K. (2012). Attitudes and acceptance of oral and parenteral HIV preexposure prophylaxis among potential user groups: A multinational study. PloS One.

[30] Zhou F., Gao L., Li S., Li D., Zhang L., Fan W., Yang X., Yu M., Xiao D., Yan L. (2012). Willingness to accept HIV pre-exposure prophylaxis among chinese men who have sex with men. PloS One.

[31] Anglemyer A., Rutherford G.W., Egger M., Siegfried N. (2011). Antiretroviral therapy for prevention of HIV transmission in hiv-discordant couples. Cochrane Database Syst. Rev..

